# Healthy Choices in Midlife Predict Survival to Age 85 in Women: The Tromsø Study 1979–2019

**DOI:** 10.3390/ijerph19095219

**Published:** 2022-04-25

**Authors:** Ola Løvsletten, Tormod Brenn

**Affiliations:** Department of Community Medicine, Faculty of Health Sciences, UiT The Arctic University of Norway, 9019 Tromsø, Norway; tormod.brenn@uit.no

**Keywords:** survival analysis, longevity, cohort, risk factors, Tromsø Study

## Abstract

The aim of this study is to examine the association between single risk factors and multiple risk factors in midlife and older ages (up to 64 years) and survival to the age of 85 years in women. The study sample comprised 857 women who attended the second survey of the population-based Tromsø Study (Tromsø2, 1979–1980) at the ages of 45–49 years and were followed for all-cause mortality until 85 years of age. Daily smoking, physical inactivity, being unmarried, obesity, high blood pressure, and high cholesterol in midlife were used as explanatory variables in survival analyses. In total, 56% of the women reached the age of 85. Daily smoking, physical inactivity, being unmarried, and obesity were significant single risk factors for death before the age of 85. None of the women had all six risk factors, but survival to age 85 did decrease gradually with increasing number of risk factors: from 67% survival for those with no risk factors to 28% survival for those with four or five risk factors. A subset of the study sample also attended the third and fourth surveys of the Tromsø Study (Tromsø3, 1986–1987 and Tromsø4, 1994–1995, respectively). Women who quit smoking and those who became physically active between Tromsø3 and Tromsø4 had higher survival when compared to those who continued to smoke and remained physically inactive, respectively. This study demonstrates the importance of having no or few risk factors in midlife with respect to longevity. We observed a substantial increase in the risk of death before the age of 85 among women who were daily smokers, physically inactive, unmarried, or obese in midlife. This risk may be mitigated by lifestyle changes, such as quitting smoking and becoming physically active later in life.

## 1. Introduction

Global life expectancy has increased over the last 150 years, and it has more than doubled since 1900 [1]. People born at the beginning of the 1800s lived an average of 30 years [1], whereas those born in 2019 have a life expectancy of 73 years [2]. Many developed countries have life expectancies above 80 years, with Japan currently ranked highest [2]; thus, longevity has reached epidemic proportions [3]. Several studies have focused on explanatory variables for longevity, and genetic factors have been found to explain approximately 25% [4]. Thus, *modifiable* risk factors (i.e., lifestyle factors) may be the most important determinants for reaching old age.

Early population-based cohort studies offer a unique possibility to study the effect of multiple risk factors in midlife on survival to advanced ages. Some of the first to conduct such a study were Terry et al., who examined the association between cardiovascular risk factors measured at the ages of 40–50 years and survival to the age of 85 in the Framingham Heart Study (FHS) [5]. The results showed the predictive power of a simple risk score, formed by counting the number of risk factors, e.g., women with all risk factors (five in that study) had a 14% chance of reaching the age of 85 compared to 65% among those with no risk factors [5]. The importance of midlife risk factors on longevity was later confirmed in studies including men only [6,7,8,9]. However, to the best of our knowledge, research on the association between number of risk factors in midlife and survival to old age in women is scarce.

The aim of this study is to examine the association between single risk factors and a number of risk factors in midlife and older age (up to 64 years) and survival to the age of 85 years in women, using data from a large population-based cohort in Norway.

## 2. Materials and Methods

### 2.1. Study Population

The Tromsø Study is an ongoing population-based cohort study which currently consists of seven surveys (Tromsø1–Tromsø7) conducted between 1974 and 2016 [10,11]. Only men were invited to Tromsø1, whereas both women and men were invited to Tromsø2 through Tromsø7. All women born in 1930–1959 with an address in the Tromsø municipality were invited to Tromsø2 in 1979–1980, and 8143 participated (81.8% attendance). All women born in 1930–1934 who attended Tromsø2 (i.e., aged 45–49 years, *n* = 868, 90.6% attendance) were eligible for inclusion in the present study. We then excluded women who withdrew their consent from the research (*n* = 4) and those who emigrated (*n* = 7), resulting in a final study sample of 857 participants.

### 2.2. Endpoint and Risk Factors

The study sample had the chance to reach age 85 prior to 2020. The participants were followed for all-cause mortality through links to the Norwegian Population Registry. Dates of birth and death were used to calculate age at death, and the endpoint was defined as age at death, censored at age 85.

Data for midlife were taken from Tromsø2. In that survey, daily smoking (yes/no), physical activity in leisure time (the Saltin-Grimby Physical Activity Level Scale, SGPALS [12]: 1-sedentary/2-moderate/3-intermediate/4-intensive) and use of blood pressure medication (yes/no) were part of a first questionnaire; blood pressure, height, and weight were measured by trained personnel; and total cholesterol was derived from a non-fasting venous blood sample. Education was categorised as >9 years (yes/no) and was taken from a second questionnaire which participants completed at home and returned by mail [13]. However, not all women who completed the first questionnaire responded to the second one [14], which led to some missing values for education (*n* = 120). We defined the SGPALS category 1 as ‘physically inactive’. Body mass index (BMI) was computed as weight in kg divided by height in m^2^, and obesity was defined as a BMI ≥30 kg/m^2^. High blood pressure was defined as use of blood pressure medication, systolic blood pressure ≥140 mmHg, or diastolic pressure ≥90 mmHg. High cholesterol was defined as total cholesterol >7 mmol/L.

Changes in daily smoking and physical inactivity in later life were evaluated using data from Tromsø3 and Tromsø4, conducted in 1986–1987 and 1994–1995, respectively. Participants who were still living in Tromsø at that time were invited to these surveys. The SGPALS was used in Tromsø3, so the same definition of physically active was applied. However, Tromsø4 did not use the SGPALS; it used two different questions on light and hard physical activity [12]. Thus, we used the question on light physical activity (response alternatives: none; <1 h/week; 1–2 h/week; ≥3 h/week) and defined ‘physically inactive’ as light physical activity <3 h/week. We defined daily smoking (yes/no) in Tromsø4 as in Løvsletten et al. [15].

### 2.3. Statistical Analysis

Descriptive statistics are presented as numbers and proportions for discrete variables, and median and interquartile ranges for continuous variables. In the survival analysis, age was used as the time variable. We used the Kaplan–Meier estimator for the survival functions and the log-rank test to test for equality between groups. To take into account the effect of differences between groups caused by other risk factors, we also applied Cox proportional hazard models with all risk factors in midlife included as covariates. Statistical tests did not reject the hypothesis of proportional hazards.

Results from the Kaplan–Meier method are presented as plots, with *p*-values from the log-rank test. We report hazard-ratios with 95% confidence intervals from the Cox models. The data analysis was performed in R [16], plots were created with ggplot2 [17], and *p* < 0.05 was considered statistically significant.

### 2.4. Ethics

This study was approved by the Regional Committee of Medical and Health Research Ethics (REK Nord 200784) and assessed by the Norwegian Centre for Research Data (NSD Data Protection Services, reference 902423).

## 3. Results

In total, 477 (56%) women from the study sample were still alive at the age of 85. Among the survivors, a lower proportion reported daily smoking, physical inactivity, being unmarried, obesity, high blood pressure, and high cholesterol in midlife than those who died prior to age 85 (Table 1).

### 3.1. Single Risk Factors

**Figure 1:**
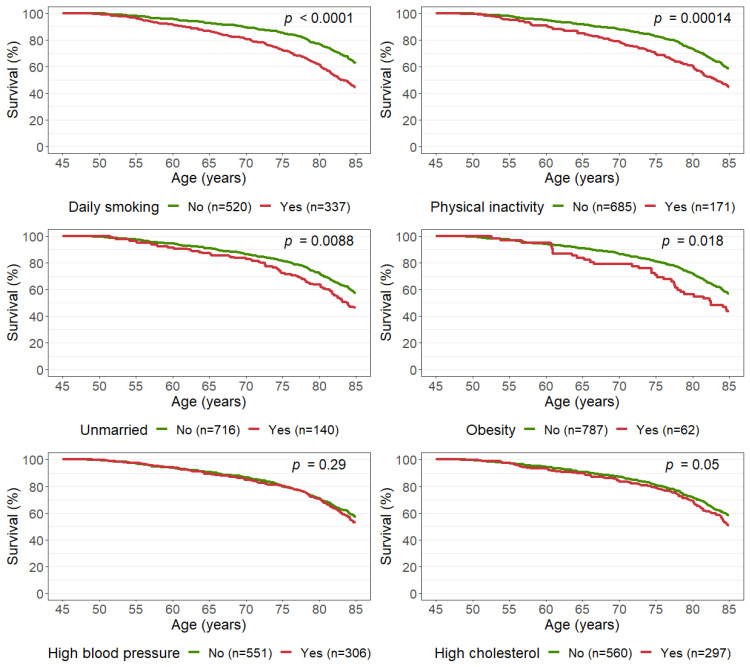
Survival curves for all-cause mortality according to risk factors observed at age 45–49 years (Tromsø2). The Tromsø Study 1979–2019. Obesity: body mass index ≥ 30 kg/m^2^; high blood pressure: blood pressure medication, systolic blood pressure ≥ 140 mmHg, or diastolic pressure ≥ 90 mmHg; high cholesterol: total cholesterol > 7 mmol/L.

Both daily smoking and physical inactivity were strong risk factors for death before age 85; 63% of those who did not report daily smoking in midlife reached age 85 compared to 45% of those who were daily smokers. Marriage and obesity were also found to be statistically significantly associated with survival to the age of 85, with unmarried and obese women having lower survival. We found no significant association between survival to age 85 and high blood pressure or high cholesterol (Figure 1). We present survival analyses that examine alternative (stricter) measures of high blood pressure and high cholesterol in the Appendix A.

The *p*-values from the multiple Cox model (Table 2) were similar to those from the unadjusted model, except perhaps for high cholesterol (*p* = 0.2 and *p* = 0.05, respectively). The direction of the associations was also the same in the multiple and unadjusted models, e.g., women who were unmarried in midlife had a higher hazard than those who were married, consistent with Figure 1. When education was included in the multiple Cox model, it did not predict survival to age 85 (*p* = 0.4). Moreover, obesity was insignificant (*p* = 0.058) in the model that included education.

### 3.2. Number of Risk Factors

**Figure 2:**
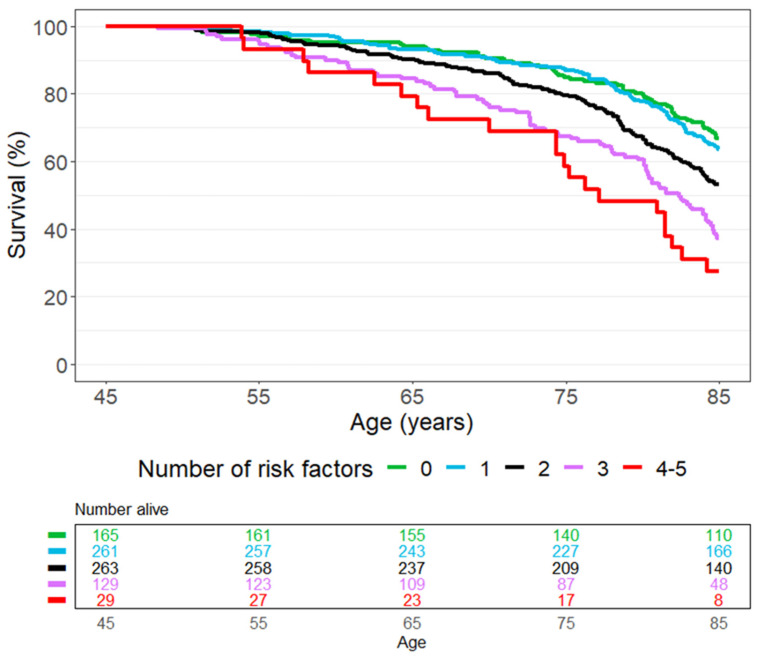
Survival curves for all-cause mortality by number of risk factors recorded at age 45–49 years (Tromsø2). The risk factors are daily smoking, physical inactivity, unmarried, obesity (body mass index ≥ 30 kg/m^2^), high blood pressure (use of blood pressure medication, systolic blood pressure ≥ 140 mmHg, or diastolic pressure ≥ 90 mmHg), and high cholesterol (total cholesterol > 7 mmol/L). The Tromsø Study 1979–2019.

None of the women in our study sample had all six risk factors (daily smoking, physical inactivity, unmarried, obesity, high blood pressure, and high cholesterol) in midlife; however, three participants had five, and 26 participants had four risk factors. Of these 29 women, 8 (28%) were alive at age 85. In contrast, 67% of women with no risk factors survived to the age of 85 (Figure 2).

### 3.3. Changes in Daily Smoking and Physical Inactivity from Midlife to Older Age

**Figure 3:**
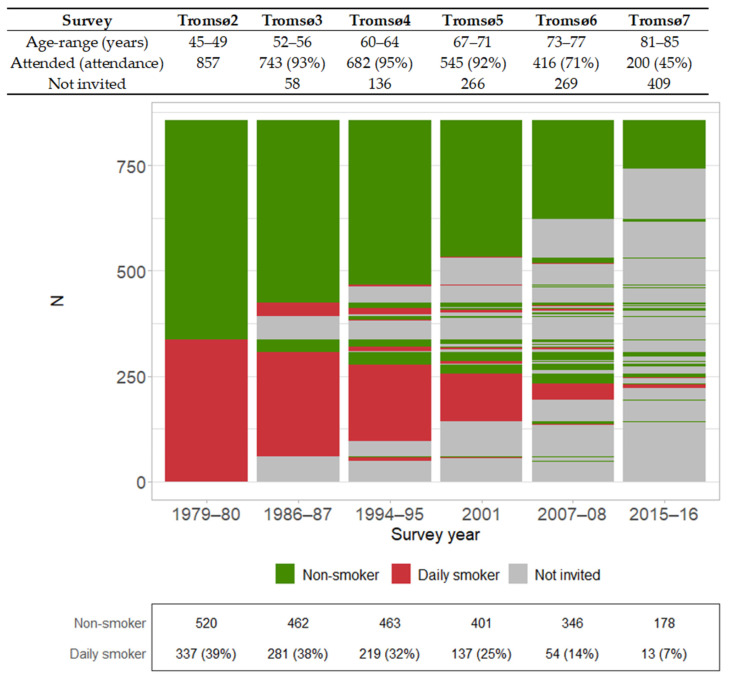
Attendance of the study sample (*N* = 857 women) to the later surveys of the Tromsø Study (top table) and lasagna plot [18] of daily smoking. The number and proportion of daily smokers are shown in the bottom table. The Tromsø Study 1974–2016. Non-smoker: did not smoke daily.

When looking at attendance and the prevalence of daily smoking across surveys, we observed differences between the number of women who attended and the sum of daily smokers and non-smokers, due to missing values (e.g., seven did not answer the daily smoking question in Tromsø5) (Figure 3).

**Figure 4:**
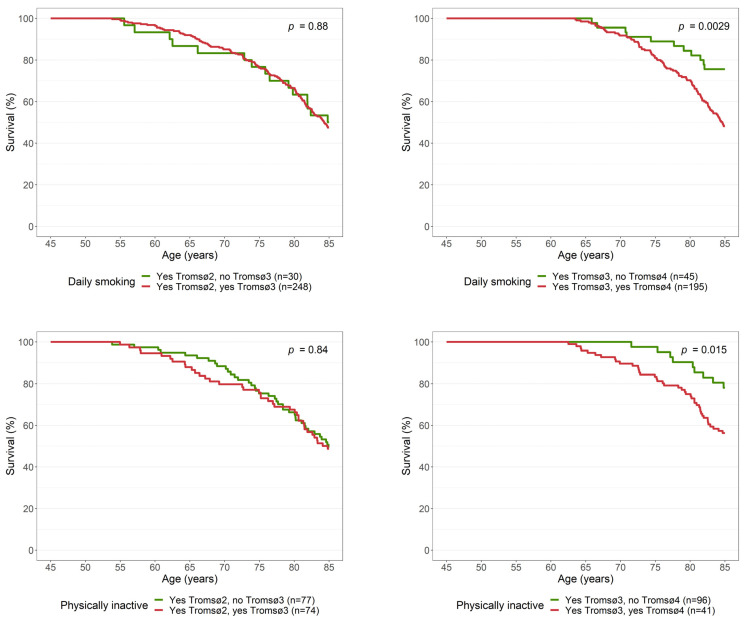
Survival curves for all-cause mortality according to change in daily smoking and physical inactivity. The Tromsø Study 1979–2019.

We observed no statistically significant difference in survival to age 85 between daily smokers who did and did not quit smoking between Tromsø2 and Tromsø3 (Figure 4). However, we did observe a difference in survival between daily smokers who did and did not quit smoking between Tromsø3 and Tromsø4, with higher survival among those who quit. A similar pattern was observed for changes in physical inactivity: no difference was observed between physically inactive women who did and did not become physically active between Tromsø2 and Tromsø3, but a significant difference was revealed between Tromsø3 and Tromsø4. Among those who reported physical inactivity at Tromsø3, those who became physically active at Tromsø4 had higher survival than those who remained physically inactive. However, it is important to note that the questions regarding physical activity were different in Tromsø4 than in Tromsø2 and Tromsø3. Adjusting for baseline covariates did not change these results (Table 3).

## 4. Discussion

We found that daily smoking, physical inactivity, being unmarried, and obesity in midlife (age 45–49 years) were significant single risk factors for death before age 85. The chance of reaching the age of 85 decreased gradually with increasing number of risk factors: from 67% for those with no risk factors to 28% for those with four or five risk factors. Those who quit smoking and those who became physically active prior to Tromsø4 (age 60–64) had higher survival than those who continued to smoke and those who remained physically inactive, respectively.

The ability of the number of risk factors (risk score) to predict survival to age 85 is in accordance with other studies that included men only [6,7] and with the FHS, which included both men and women [5]. However, the variables considered differed between this study and the FHS, which may be partly explained by different methodologies. In the FHS, stepwise model selection was used to determine which variables to include in the risk score; these ultimately included sex, systolic blood pressure, serum cholesterol, glucose intolerance, smoking history, and education. In contrast, we chose six risk factors a priori and included total cholesterol, but not glucose intolerance. This choice was partly based on what was found to predict survival to age 90 in men in The Tromsø Study [6]. Also, except for marital status, these risk factors are some of the established risk factors for death identified in women in the Global Burden of Disease (GBD) project, though the GBD moved from estimating total cholesterol in 2016 to estimating LDL cholesterol in 2017 [19].

A physical activity index was considered in the FHS but was not included in the final (most parsimonious) model. In contrast, we found that being physically active was a significant predictor of survival and longevity, consistent with other studies [20]. Another difference between our results and those of the FHS is that we did not observe any effect of high systolic blood pressure on survival, and we saw a non-significant association with total cholesterol. In contrast to our findings, the GDB project ranked systolic blood pressure as the number one risk factor for death in women [19]. However, further analyses of our data (presented in the Appendix A) showed an association between a stricter definition of high blood pressure and survival.

Though women who were daily smokers in midlife had a lower chance of reaching age 85, the proportion who did so was still 45%. This relatively high number of survivors can be explained by the fact that many quit daily smoking later in life (Figure 3). This higher survival among quitters is consistent with previous findings from the Netherland Cohort Study [21]. However, that study had the limitation of using baseline information to define quitters due to lack of follow-up surveys, whereas we were able to use information from later surveys. We observed no improvement in survival time among daily smokers who did and did not quit smoking between Tromsø2 and Tromsø3, which is similar to what was observed previously for men [6]. This phenomenon may be difficult to comprehend and we have no explanation for these null findings; however, we did observe that quitters had significantly higher survival than persistent smokers when we compared Tromsø2 and Tromsø4 and later surveys. We interpret this as strong evidence that quitting smoking increases the probability of survival.

As previously found for men in the Tromsø Study [6], we observed that women who were married in midlife had higher survival than those who were unmarried, a result that is in accordance with other studies [22]. Unlike other studies [5,22], we did not find a significant effect of education. However, the risk factor of obesity became insignificant when education was included in the model. A similar finding was reported for BMI in Terry et al. [5].

All six of the investigated risk factors are modifiable, thus individuals can make lifestyle changes in midlife or later in life that may improve their longevity. We consider this to be an important message to communicate to the public. At the same time, lasting lifestyle changes can be difficult to achieve without the support of community-based interventions. For instance, since 1975, various legislative measures were implemented in Norway, which is a likely cause for the large decline in cigarette smoking [15].

Strengths of this study include the prospective study design with a follow-up time of 40 years, anthropometric measurements that were performed by trained personnel, and objective measurements obtained from blood samples. Another strength is the population-based sample and the high attendance rate among women aged 45–49 years (90.6%) in Tromsø2 and subsequent surveys (Figure 3).

We did not include morbidity-free survival in this analysis, which may be considered a limitation, as increased attention has been placed on healthy aging. However, previous knowledge suggests that factors that promote longevity are highly correlated with delayed morbidity [23]. Another limitation is the observational study design, which means that we cannot claim causality, only associations. Finally, there are other risk factors that may be of importance to reach longevity. Thus, our conclusion comes with the limitation that only six risk factors were considered.

## 5. Conclusions

This study demonstrates the importance of having no, or few, risk factors in midlife with respect to longevity. One should refrain from smoking and be physically active. For those who smoke or are physically inactive in midlife, quitting smoking and becoming physically active is likely to increase life expectancy.

## Figures and Tables

**Table 1:** Descriptive statistics of risk factors in the study sample at age 45–49 years (Tromsø2), according to survival to age 85. The Tromsø Study 1979–2019.

Characteristic	Overall *N* = 857 ^1^	Alive at Age 85 *N* = 477 ^1^	Died before Age 85 *N* = 380 ^1^
Daily smoking	337 (39%)	150 (31%)	187 (49%)
Physical inactivity	171 (20%)	76 (16%)	95 (25%)
Unmarried	140 (16%)	65 (14%)	75 (20%)
Body mass index (kg/m^2^)	23.6 (21.8, 26.0)	23.5 (21.8, 25.8)	23.7 (21.8, 26.4)
Obesity	62 (7.3%)	27 (5.7%)	35 (9.3%)
High blood pressure	306 (36%)	162 (34%)	144 (38%)
Blood pressure medication	43 (5.0%)	18 (3.8%)	25 (6.6%)
Systolic blood pressure (mmHg)	126 (118, 138)	126 (116, 138)	127 (118, 140)
Diastolic blood pressure (mmHg)	82 (76, 90)	82 (76, 88)	84 (78, 90)
Total cholesterol (mmol/L)	6.50 (5.75, 7.39)	6.44 (5.71, 7.32)	6.65 (5.79, 7.51)
High cholesterol	297 (35%)	151 (32%)	146 (38%)

^1^*n* (%); Median (interquartile range). Obesity: body mass index ≥ 30 kg/m^2^; high blood pressure: use of blood pressure medication, systolic blood pressure ≥ 140 mmHg, or diastolic pressure ≥ 90 mmHg; high cholesterol: total cholesterol > 7 mmol/L.

**Table 2:** Hazard ratios for all-cause mortality from a multiple Cox model with risk factors observed at ages 45–49 years (Tromsø2). The Tromsø Study 1979–2019.

Risk Factors	Hazard Ratio	95% Confidence Interval	*p*-Value
Daily smoking	1.77	1.44, 2.19	<0.001
Physical inactivity	1.43	1.12, 1.82	0.004
Unmarried	1.37	1.06, 1.78	0.015
Obesity	1.67	1.17, 2.38	0.005
High blood pressure	1.16	0.94, 1.44	0.2
High cholesterol	1.17	0.95, 1.44	0.2

Obesity: body mass index ≥ 30 kg/m^2^; high blood pressure: blood pressure medication, systolic blood pressure ≥ 140 mmHg, or diastolic pressure ≥ 90 mmHg; high cholesterol: total cholesterol > 7 mmol/L.

**Table 3:** Hazard ratios for all-cause mortality from a multiple Cox model. The Tromsø Study 1979–2019.

Characteristic	Hazard Ratio	95% Confidence Interval	*p*-Value
Quitter ^1^ (Tromsø2 and Tromsø3)	1.07	0.62, 1.83	0.8
Quitter ^1^ (Tromsø3 and Tromsø4)	0.41	0.22, 0.78	0.006
Physically inactive → active ^2^ (Tromsø2 and Tromsø3)	1.24	0.76, 2.05	0.4
Physically inactive → active ^2^ (Tromsø3 and Tromsø4 ^3^)	0.44	0.21, 0.92	0.029

Adjusted for daily smoking, physical inactivity, being unmarried, obesity, high blood pressure, and high cholesterol at age 45–49 years (Tromsø2). ^1^ Quitter = Smoked daily in first survey and did not smoke daily in second survey. Reference level: daily smoker in both surveys. ^2^ Reference level: physically inactive at both surveys. ^3^ Different question than in Tromsø2 and Tromsø3; in Tromsø4, physically inactive was defined as <3 h/week of light activity.

**Figure A1:**
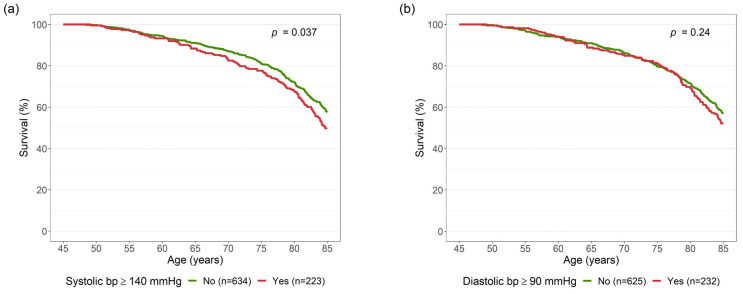
Survival curves by high systolic bp (**a**) and high diastolic bp (**b**). Those who reported use of bp medication are included in the yes categories. The Tromsø Study 1979–2019. bp: blood pressure.

**Figure A2:**
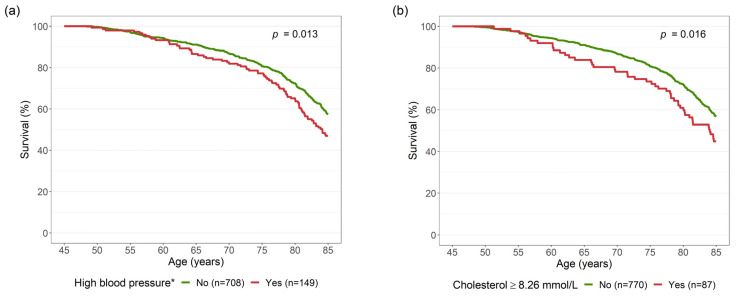
Survival curves by high blood pressure * (**a**) (systolic blood pressure ≥ 140 mmHg and diastolic blood pressure ≥ 90 mmHg, or blood pressure medication) and high cholesterol (**b**) (≥8.26 mmol/L, i.e., the 90% quantile). The Tromsø Study 1979–2019.

## Data Availability

Researchers may apply for access to data, see instructions at www.tromsostudy.com [14].

## Appendix A

### High Blood Pressure and High Cholesterol

In the main analysis, we found that the definition of high blood pressure (BP, use of BP medication, systolic BP ≥ 140 mmHg, or diastolic BP ≥ 90 mmHg) was not significantly associated with survival to age 85 years. To some degree, this was also the case for high cholesterol (*p* = 0.05 in the unadjusted model, *p* = 0.2 in the multiple Cox model). Therefore, here we examine alternative measures of high BP and high cholesterol.

A cross tabulation of the number of participants with systolic BP ≥140 mmHg and diastolic BP ≥90 mmHg are presented in Table A1. A total of 11 participants did not have diastolic BP ≥90 mmHg but reported using BP medication, and the same number of participants (11) did not have systolic BP ≥140 mmHg but reported using BP medication. We observed that systolic BP ≥140 mmHg or use of BP medication had a significant effect on survival, whereas diastolic BP ≥90 mmHg did not (Figure A1). Therefore, we applied a stricter definition of high blood pressure: systolic BP ≥140 mmHg and diastolic BP ≥90 mmHg, or BP medication (in Figure A2a). For cholesterol we considered the 90% quantile (Figure A2b). Results from the multiple Cox model are presented in Table A2.

**Table A1:** Number with high systolic and diastolic blood pressure at age 45–49 years (Tromsø 2). The Tromsø Study 1979–1980.

	Diastolic Blood Pressure ≥ 90 mmHg		
Systolic Blood Pressure ≥ 140 mmHg	No	Yes	Total
No	560	85	645
Yes	76	136	212
Total	636	221	857

**Table A2:** Hazard ratios for all-cause mortality from a multiple Cox model with risk factors observed at age 45–49 years (Tromsø2). The Tromsø Study 1979–2019.

Risk Factor	Hazard Ratio	95% Confidence Interval	*p*-Value
Daily smoking	1.78	1.45, 2.19	<0.001
Physical inactivity	1.41	1.11, 1.79	0.005
Unmarried	1.36	1.05, 1.76	0.018
Obesity	1.79	1.20, 2.69	0.004
High blood pressure *	1.36	1.06, 1.76	0.017
High cholesterol **	1.30	0.96, 1.77	0.094

Obesity: body mass index ≥ 30 kg/m^2^; high blood pressure *: systolic blood pressure ≥ 140 mmHg and diastolic blood pressure ≥ 90 mmHg, or blood pressure medication; high cholesterol **: ≥8.26 mmol/L, i.e., the 90% quantile.
